# How to design clinical rehabilitation trials for the upper paretic limb early post stroke?

**DOI:** 10.1186/s13063-016-1592-x

**Published:** 2016-09-26

**Authors:** Caroline Winters, Martijn W. Heymans, Erwin E. H. van Wegen, Gert Kwakkel

**Affiliations:** 1 Department of Rehabilitation Medicine, VU University Medical Center, MOVE Research Institute Amsterdam, Amsterdam, The Netherlands; 2 Neuroscience Campus Amsterdam, Vrije Universiteit, Amsterdam, The Netherlands; 3 Department of Epidemiology and Biostatistics, VU University Medical Center Amsterdam, Amsterdam, The Netherlands; 4 Department of Methodology and Applied Biostatistics, Faculty of Earth and Life Sciences, Vrije Universiteit, Amsterdam, The Netherlands; 5 Amsterdam Rehabilitation Research Center, Reade, Amsterdam, The Netherlands; 6 Department of Physical Therapy and Human Movement Sciences, Northwestern University, Chicago, IL USA

**Keywords:** Stroke, Randomized controlled trial, Power, Sample size, Prognostic stratification, Upper extremity

## Abstract

**Background:**

The impact of spontaneous neurobiological recovery is still neglected in designing rehabilitation trials early post stroke. We aimed to investigate the impact of the timing of randomization and prognostic stratification on the required sample sizes that are needed to reveal significant intervention effects on upper limb function at 26 weeks after first-ever ischemic stroke.

**Method:**

Sample size calculations were based on a cohort study of 159 patients, using the Fugl-Meyer Assessment Upper Extremity and Action Research Arm Test as outcome measures (power = 80 %; two-tailed alpha = 0.05). We investigated different scenarios: random sampling of patients within five time intervals (stroke onset to 1, 3, 5, 8 and 12 weeks post stroke), and within stratified groups according to the presence or absence of voluntary extension of the thumb and/or two or more fingers at intake.

**Results:**

The heterogeneity between outcome scores of patients, and subsequently the required sample sizes, increased from the first to the fifth time interval. Compared to the whole group, the sample sizes for both stratified groups (i.e., patients with and without Voluntary Finger Extension (VFE)) were lower. The required sample sizes for the patient group without VFE markedly increased when the time interval was broadened from 1 to 12 weeks post stroke, as opposed to the decrease seen for the group of patients with VFE.

**Conclusion:**

These results are fundamental for designing upper limb trials early post stroke. To prevent type II error, future upper limb trials should randomize patients at a fixed moment early post stroke and stratify patients according to their potential neurobiological recovery.

**Trial registration:**

Netherlands Trial Registry, www.trialregister.nl, NTR1424, registered on 27 August 2008.

## Background

Recent systematic reviews and meta-analyses of stroke rehabilitation trials that are started early after stroke show that the effect sizes of interventions are small to moderate and account for 5 to 10 % of the differences in outcome [1, 2]. Approximately 98 % of all randomized controlled trials (RCTs) are proof-of-concept trials and are often heavily underpowered [1]. At this moment, there is no evidence that stroke rehabilitation programs started within the first 3 months post stroke are more effective than programs initiated beyond this time period, despite the growing evidence of heightened brain plasticity early post stroke [3–5].

Only about 7 % (*N* = 18) of the clinical stroke trials that focus on upper limb recovery performed the randomization procedure within the first 2 weeks after stroke onset (i.e., hospital-based trials) [1]. The majority of RCTs start their randomization procedure when patients are discharged from the hospital and admitted to a rehabilitation ward or nursing home [1]. As a consequence, inclusion of subjects in most phase II trials ranges from a few days up to several months post stroke. Such pragmatic design of RCTs ignores the impact of spontaneous neurobiological recovery during the first 5 to 10 weeks post stroke which accounts for about 80 % of all neurological improvement that is clinically observed in longitudinal cohort studies of the upper limb, lower limb and cognitive impairments [6–9]. One may, therefore, raise the fundamental question whether the timing of randomization in the first 12 weeks post stroke is an important factor for designing phase II trials in stroke rehabilitation. One may hypothesize that the arbitrary timing of randomization post stroke causes type II errors in small RCTs through the additional variance that is introduced by still poorly understood, time-dependent processes of spontaneous neurobiological recovery early post stroke.

Furthermore, several systematic reviews and meta-analyses suggest that evidence-based therapies for the upper paretic limb are strongly dependent on an appropriate selection of patients at baseline [10, 11]. Several prospective cohort studies showed that the ability to voluntarily extend one or more fingers against gravity within the first 3 days is a robust clinical marker for upper limb recovery after 3 or 6 months post stroke [12, 13], reflecting the intactness of the corticospinal tract [14]. Unfortunately, only one clinical trial out of the 266 upper limb trials published [1] stratified patients on the basis of their initial impairment prior to the randomization procedure [15]. At this moment, evidence-based interventions seem to be restricted to those patients with some Voluntary Finger Extension (VFE) [1, 16, 17]. One may hypothesize that the choice of whether or not to stratify patients prior to randomization based on early prognosis, for example using VFE, influences the heterogeneity in upper limb function between patients and consequently the probability of finding differential effects post intervention [6, 10, 18].

The aims of the present study were to investigate the impact of (1) different time intervals that vary in length between stroke onset and randomization and (2) prognostic stratification based on the presence or absence of VFE, on the required sample size needed to reveal significant and clinically important intervention effects on the Action Research Arm Test (ARAT) and Upper Extremity motor section of the Fugl-Meyer Assessment (FMA-UE) at 26 weeks after stroke.

## Methods

### Study population and procedure

Data from the EXPLCIT-stroke trial were used [16]. Details of this RCT can be found elsewhere [16, 19]. The inclusion criteria were: (1) first-ever middle cerebral aftery ischemic stroke, (2) upper limb paresis according to item 5 of the National Institutes of Health Stroke Scale (NIHSS >1 point), (3) Mini Mental State Examination score ≥23 points, (4) age between 18 and 80 years, (5) no upper limb musculoskeletal impairments, (6) no botulinum toxin treatment in the previous 3 months, (7) the ability to sit independently for 30 s and (8) provision of written informed consent.

At intake within 2 weeks post stroke, patients were stratified to (1) a group of patients presenting with VFE (*N* = 58) and randomly assigned to either modified Constraint-induced Movement Therapy (mCIMT) or usual care or (2) a group of patients presenting without VFE (*N* = 101) and randomly assigned to electromyogram (EMG)-triggered Neuro-muscular Stimulation (EMG-NMS) or usual care. The patients with VFE had the ability to voluntarily extend the thumb and/or two or more fingers of the affected hand (to 10° or more). The functional assessments were repeated weekly up to 5 weeks after stroke and at 8-, 12- and 26-week follow-ups [16, 19].

### Outcome measurements

In the present study we used the ARAT and FMA-UE as primary outcome measures. The ARAT is an upper limb capacity test which assesses the ability to grasp, move and release objects of various sizes, weights and shapes. It has 19 subquestions scored on a 4-point ordinal scale, adding up to a total score between 0 and 57 points (57 = normal capacity) [20, 21]. The Minimal Clinically Important Difference (MCID) was set at 5.7 points, i.e., 10 % of the range [22]. The FMA-UE assesses limb impairment in terms of synergistic motor control. It has 22 subquestions scored on a 3-point ordinal scale, adding up to a total score between 0 and 66 points (66 = normal function) [23, 24]. The MCID was set at 6.6 points [25, 26].

### Statistical analysis

Approximately 8 % of the 1272 assessments in the EXPLICIT-stroke trial were missing due to various reasons (recurrent stroke, sickness, etc.) [16]. We estimated these missing data points using individual curve fitting for subjects with two or more available assessments by estimating the ARAT and FMA-UE recovery curves using a linear mixed model (linear and quadratic component) that best described the individual recovery pattern, and that accounted for the repeated measures. The estimated data were merged with the original data to create a new complete dataset and checked by visual inspection. All further analyses were performed on this new (modelled) dataset. A total of 157 out of the 159 patients were eligible for further analysis: one patient had only one available assessment and another patient’s recovery was negatively influenced by open heart surgery 4 months post stroke.

For the first aim we randomly varied the length of the time interval from stroke onset to randomization for each patient. Five different time intervals were evaluated: stroke onset (T_0_) to 1 week post stroke, T_0_ to 3 weeks, T_0_ to 5 weeks, T_0_ to 8 weeks and T_0_ to 12 weeks. Within each time interval patients (*N* = 157) were randomly selected, resulting in a dataset in which some patients were included with a follow-up measurement at 1 week, others at 2, 3, 4 or 5 weeks when the time interval of 5 weeks after stroke onset was used. In this way heterogeneity in recruitment period post stroke onset was guaranteed.

For the second aim we adopted the EXPLICIT-stroke trial patient allocation to either the group of patients with or without VFE at intake. Fifty-seven patients with VFE at intake were available for analysis (with one dropout as described above). To obtain equal groups, we randomly selected 57 out of the 100 patients without VFE at intake using random sampling in SPSS (version 22), taking into account the distribution of randomization.

The minimum number of subjects in each group that is needed to find a differential effect at 26-week follow-up was calculated using Eq. 1. This number per group was multiplied by 2 to obtain the total number of subjects where after 10 % was added to account for dropouts. We used a standard *t* test sample size calculation to assess group differences at 26-week follow-up, assuming a normally distributed outcome. The power was set at 80 % and two-tailed alpha at 0.05. The standard deviation (SD) was determined using randomly selected patients for each different post stroke time interval as explained above. Different scenarios were used, selecting: (1) all patients (*N* = 157), (2) a subgroup of 114 patients including 57 patients with VFE and 57 patients without VFE, (3) patients with VFE at intake (*N* = 57) and (4) patients without VFE at intake (*N* = 57), within the five different time intervals. The average of the SD of the two groups (Eq. 3) was used to calculate the Cohen’s *d* effect size (Eq. 2).

1 $$ {N}_{group}=2\times \frac{{\left[{Z}_{\alpha }+{Z}_{\beta}\right]}^2}{d^2} $$

2 $$ d=\frac{{\overline{x}}_1-{\overline{x}}_2}{S{D}_{pooled}} $$

3 $$ S{D}_{pooled}=\frac{\sqrt{S{D}_1^2 + S{D}_0^2}}{2} $$

Where *N*_*group*_ is the number of subjects per group; *Z*_*β*_ = 0.842; *Z*_*α*_ = 1.96; *d =* Cohen’s effect size; ${\overline{x}}_1-{\overline{x}}_2$ = group mean difference at 26-week follow-up. This difference was set at the MCID of the ARAT and FMA-UE, respectively 5.7 and 6.6 points; *SD*_*pooled*_ = the average of the standard deviation of the sample (full dataset, after individual curve fitting); and *SD*_1_ and *SD*_0_ = standard deviation for, respectively, the intervention and the control group (full dataset, after individual curve fitting).

The *SD*_*pooled*_ values are presented as variances. Patients received an intervention after randomization. We therefore recalculated the sample size estimations by taking account of this intervention effect by deriving the *SD*_*pooled*_ from a linear regression model that included the intervention group variable, with as outcome the FMA-UE and ARAT score. The (square root of the) unexplained variances from this model were used as the *SD*_*pooled*_, “controlled for” the intervention effect. As these were the same as the raw *SD*_*pooled*_ values, we will present sample size estimations using the raw *SD*_*pooled*_ values. Analyses were performed using R (version 3.1.1), unless otherwise indicated.

## Results

**Fig. 1:**
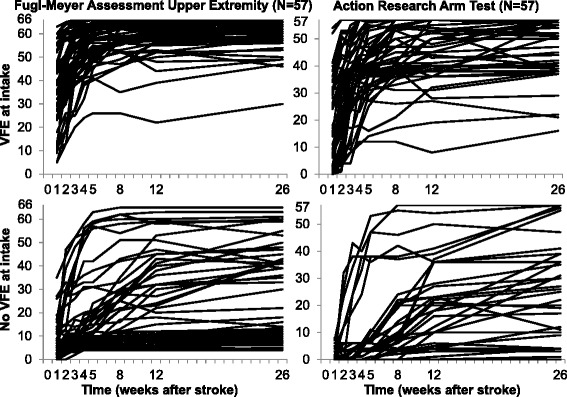
Individual FMA-UE and ARAT recovery curves for patients with and without Voluntary Finger Extension (VFE). The top two graphs represent the patients with VFE at about 1 week after stroke and the bottom two graphs the patients without VFE. The lefthand two graphs and righthand two graphs represent, respectively, the Upper Extremity motor scores of the Fugl-Meyer Assessment (FMA-UE, score = 0–66, 66 = normal function) and the Action Research Arm Test (ARAT, score = 0–57, 57 = normal capacity)

Table 1 shows the characteristics of the 157 patients included in the present study. The “individual curve fitting” method was found to be successful after visual inspection of the individual recovery curves on the FMA-UE and ARAT. The FMA-UE and ARAT recovery curves are presented in Fig. 1.

**Table 1:** Patient characteristics

Determinants	*N* = 157
Sex, male/female (*N*)	94/63
Age, years (mean ± SD)	59.9 ± 9.3
Affected hemisphere, right/left (*N*)	140/53
Bamford classification, LACI/PACI/TACI (*N*)	96/52/9
Barthel Index (0–20 points, median (interquartile range))	9 (5–13)

*LACI* lacunar anterior cerebral infarction, *PACI* partial anterior cerebral infarction, *SD* standard deviation, *TACI* total anterior cerebral infarction

**Fig. 2:**
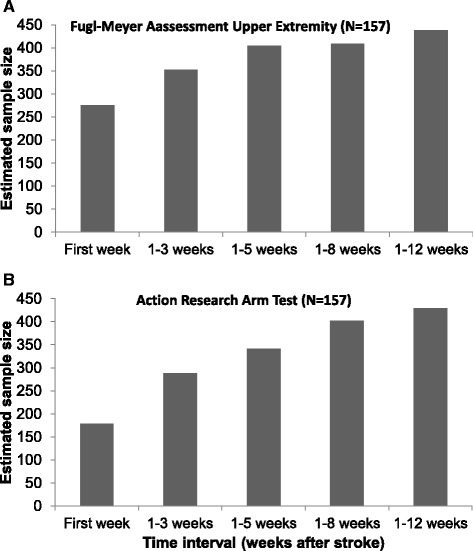
Impact of the timing of randomization on the sample size. **a** Upper Extremity motor section of the Fugl-Meyer Assessment, *N* = 157. **b** Action Research Arm Test, *N* = 157

Changing the time interval between stroke onset and randomization showed an increase in the required sample size to obtain an effect beyond MCID from the first to fifth time intervals for 148 subjects for the FMA-UE and 228 subjects for the ARAT (Table 2 and Fig. 2). The largest increase was visible between the first two time intervals for both outcome measures (ΔFMA-UE_T1-T2_ = 77 subjects and ΔARAT_T1-T2_ = 110 subjects).

**Table 2:** Results sample size calculation: various time intervals from stroke onset to the moment of randomization (*N* = 157)

Time interval	Group 1 Mean ± SD	Group 0 Mean ± SD	SD pooled	Variance	Sample size
FMA-UE					
T_0_ to 1 week	18.09 ± 19.78	15.62 ± 17.06	18.47	341	275
T_0_ to 3 weeks	22.19 ± 21.86	21.23 ± 20.07	20.98	440	352
T_0_ to 5 weeks	28.89 ± 23.42	24.27 ± 21.66	22.56	509	405
T_0_ to 8 weeks	24.54 ± 22.91	25.80 ± 22.36	22.64	512	409
T_0_ to12 weeks	26.18 ± 24.19	28.69 ± 22.61	23.42	548	438
ARAT					
T_0_ to 1 week	8.24 ± 12.82	7.33 ± 12.89	12.85	165	178
T_0_ to 3 weeks	11.70 ± 16.76	11.15 ± 15.96	16.36	268	288
T_0_ to 5 weeks	13.58 ± 17.67	13.77 ± 17.95	17.81	317	341
T_0_ to 8 weeks	15.65 ± 19.76	15.14 ± 19.02	19.39	376	403
T_0_ to 12 weeks	17.95 ± 21.49	15.46 ± 18.34	19.98	399	429

Sample sizes are the total number of patients required, including 10 % to account for dropouts. Individual patients were randomly selected at different time points post stroke onset, where after this assessment was considered as their baseline assessment. Mean and SD are derived from the full dataset, after individual curve fitting. *ARAT* Action Research Arm Test (score = 0–57, 57 = normal capacity), *FMA-UE* Upper Extremity motor section of the Fugl-Meyer Assessment (score = 0–66, 66 = normal function), *SD* standard deviation, *T*_*0*_ stroke onset

**Fig. 3:**
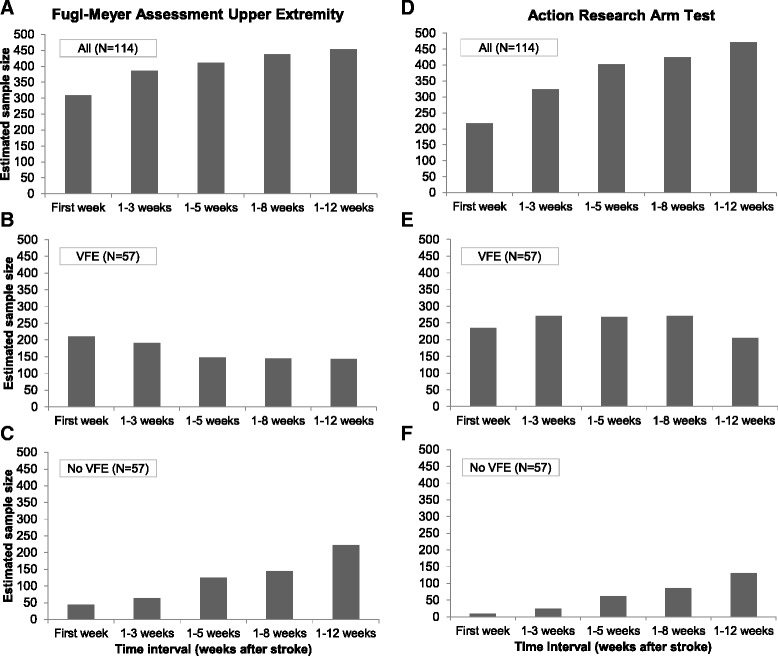
Impact of prognostic stratification based on Voluntary Finger Extension (VFE) on the sample size. **a** Upper Extremity motor section of the Fugl-Meyer Assessment (FMA-UE), all patients (*N* = 114). **b** FMA-UE, patients with VFE (*N* = 57). **c** FMA-UE, patients without VFE (*N* = 57). **d** Action Research Arm Test (ARAT), all patients (*N* = 114). **e** ARAT, patients with VFE (*N* = 57). **f** ARAT, patients without VFE (*N* = 57)

When patients were not stratified based on VFE, the required sample size for the first time interval (i.e., T_0_ to 1 week post stroke) was 308 and 218 subjects for, respectively, the FMA-UE and ARAT (Table 3 and Fig. 3). In comparison to the whole group (*N* = 114), the required sample sizes for the group of patients without VFE were lower; respectively, 44 and 9 subjects for the FMA-UE and ARAT. For the group of patients with VFE, the required sample size for the FMA-UE was also lower in comparison to the whole group (211 subjects). The required sample size with the ARAT as outcome measure was slightly higher due to a greater heterogeneity between patients (235 subjects; see also the individual recovery patterns in Fig. 1).

**Table 3:** Results sample size calculation: stratification based on presence or absence of voluntary finger extension

	All patients (*N* = 114)					Voluntary Finger Extension (*N* = 57)					No Voluntary Finger Extension (*N* = 57)				
Time interval	Group 1 Mean ± SD	Group 0 Mean ± SD	SD pooled	Variance	Sample size	Group 1 Mean ± SD	Group 0 Mean ± SD	SD pooled	Variance	Sample size	Group 1 Mean ± SD	Group 0 Mean ± SD	SD pooled	Variance	Sample size
FMA-UE															
T_0_ to 1 week	23.17 ± 20.80	19.73 ± 18.36	19.61	385	308	39.31 ± 16.59	33.68 ± 15.74	16.17	262	211	7.03 ± 8.11	5.79 ± 5.92	7.10	50	44
T_0_ to 3 weeks	29.22 ± 23.10	26.21 ± 20.61	21.89	476	385	45.55 ± 15.19	42.50 ± 15.62	15.40	237	191	9.62 ± 9.62	7.86 ± 7.84	8.77	77	64
T_0_ to 5 weeks	32.05 ± 23.61	30.57 ± 21.73	22.69	515	411	50.24 ± 14.88	48.46 ± 11.85	13.45	181	147	9.97 ± 11.32	13.21 ± 13.39	12.40	154	125
T_0_ to 8 weeks	30.83 ± 24.00	33.36 ± 22.76	23.39	547	438	49.41 ± 13.80	49.75 ± 12.90	13.36	178	145	12.38 ± 12.98	11.64 ± 13.79	13.39	179	145
T_0_ to 12 weeks	34.33 ± 25.09	31.09 ± 22.44	23.80	567	453	51.03 ± 14.22	51.29 ± 12.14	13.22	175	143	16.24 ± 17.04	16.04 ± 16.15	16.60	276	222
ARAT															
T_0_ to 1 week	10.91 ± 13.98	9.91 ± 14.39	14.19	201	218	20.62 ± 14.06	19.21 ± 15.48	14.79	219	235	1.21 ± 2.29	0.61 ± 1.69	2.01	4	9
T_0_ to 3 weeks	16.16 ± 17.60	14.98 ± 17.14	17.37	302	323	29.07 ± 16.06	27.54 ± 15.62	15.84	251	271	2.48 ± 6.10	0.82 ± 1.98	4.54	21	24
T_0_ to 5 weeks	18.76 ± 19.72	18.21 ± 19.08	19.40	376	403	34.35 ± 16.45	28.36 ± 15.13	15.80	250	268	2.55 ± 6.75	3.61 ± 7.89	7.34	54	62
T_0_ to 8 weeks	22.22 ± 20.85	17.20 ± 19.01	19.95	398	425	34.93 ± 16.57	32.00 ± 15.18	15.89	253	271	5.07 ± 10.48	3.71 ± 6.77	8.82	78	86
T_0_ to 12 weeks	22.33 ± 22.04	21.48 ± 19.80	20.95	439	471	38.17 ± 13.31	35.79 ± 14.12	13.72	188	205	4.03 ± 9.14	5.61 ± 12.39	10.89	119	130

Sample sizes are the total number of patients required, including 10 % to account for dropouts. Individual patients were randomly selected at different time points post stroke onset, where after this assessment was considered as their baseline assessment. Mean and SD are derived from the full dataset, after individual curve fitting. *ARAT* Action Research Arm Test (score = 0–57, 57 = normal capacity), *FMA-UE* Upper Extremity motor section of the Fugl-Meyer Assessment (score = 0–66, 66 = normal function), *SD* = standard deviation, *T*_*0*_ stroke onset

The required sample sizes for the group of patients without VFE increased when the time interval between stroke onset and randomization was broadened. The largest sample size was found for the broadest time interval, i.e., when randomization was performed between stroke onset and 12 weeks post stroke. For the group of patients with VFE at intake, with the FMA-UE as outcome measure, we found a progressive decrease in sample size when the time interval was broadened. For the ARAT, we observed an increase in the sample sizes when the time interval was broadened from 3 to 5 weeks, after which it remained constant before decreasing to 205 subjects in the fifth time interval. The required sample sizes for the groups of patients with and without VFE separately remained lower in comparison to the whole group throughout the second to fifth time intervals.

## Discussion

The aim of the present study was to investigate the impact of the timing of randomization and prognostic stratification on the required sample size to reveal significant and clinically important intervention effects on the FMA-UE and ARAT at 26-week follow-up. We used different scenarios for random patient recruitment based on data from a recently published RCT with repeated measuments [16]. We were able to show that the timing of the moment of randomization post stroke, and stratification based on the prognostic determinant VFE, are fundamental for preventing type II errors in neurorehabilitation trials post stroke. This finding is in agreement with the study by Duncan and coworkers [6] who also showed that the length of time from stroke onset (baseline, 5-day or 30-day status) and the severity of motor impairment measured with the FMA motor score (mild to severe) influences the chance of showing 50 % improvement in the residual motor deficit. In the present study, randomization of patients at arbitrary time points post stroke (i.e., wide time intervals) showed a tremendous increase in the required sample size. The results underpin the importance of carefully designing future RCTs to increase the chance of finding differential intervention effects. At present, upper limb trials with 2 experimental arms, assuming 80 % statistical power with randomization at a fixed moment in the first 3 months after stroke, are lacking in the scientific literature.

Lack of prognostic stratification would give an incomplete representation of the changes in underlying subgroups. Interestingly, the timing of the moment of randomization and stratification of subjects in upper limb trials are not independent phenomena for estimation of the number of subjects required for sufficiently powered rehabilitation trials. In the stratified group of patients with VFE within the first week, we observed a slight, overall decrease in the heterogeneity between outcome scores of patients when the time interval between stroke onset and randomization was increased. After 3 months, the group of mild to moderately impaired patients was more homogenous and, as a consequence, significant smaller sample sizes were required to find clinical meaningful effects of 10 % on the FMA-UE or ARAT.

The heterogeneity between outcome scores of patients in the stratified group of patients with severe motor impairments (i.e., no VFE) was low when randomization occurred in the first few weeks after stroke onset. Thereafter, the heterogeneity between patients increased substantially when the time interval for randomization was extended to 8 or 12 weeks post stroke. As previous studies showed, severely impaired patients will most likely reach their plateau in motor recovery later in time in comparison to mild to moderately impaired patients [6, 7]. The majority of these severely impaired patients will only show minimal improvement in upper limb function and there are still no evidence-based interventions for this specific group of patients [1, 11, 16]. However, a portion of the severely impaired patients in the current dataset displayed more recovery of upper limb function than predicted (i.e., mainly “false negatives”), which markedly increased the heterogeneity in outcome scores between patients when the time intervals for randomization were broadened [12].

Recent prospective cohort studies have suggested that the amount of spontaneous neurobiological recovery is highly predictable within the first 72 h after stroke onset, and that the majority of patients will recover to a level of about 70 to 80 % of their maximum possible improvement based on their initial impairment [14, 27–30]. At present, we can only assign patients retrospectively and discriminate between “fitters” and “non-fitters” in terms of expected spontaneous neurobiological recovery. There is a need for prospective stratification of patients according to their potential neurobiological recovery determined early after stroke [14]. Therefore, prognostic biomarkers are needed to identify patients who will and will not show the expected spontaneous biological recovery next to robust clinical markers such as VFE. In addition, we are of the opinion that the above recommendations with respect to the timing of randomization procedures and applying stratification in designing upper limb trials are not unique for upper limb trials. In particular, acknowledging that outcomes of the lower paretic limb parallel those of the upper limb [6, 7] and that the prognosis of meaningful outcomes, such as walking ability, is strongly dependent on initial sitting balance and lower limb strength [31]. In addition, there is growing evidence that this maximum proportional recovery rule of spontaneous neurobiological recovery is not restricted to motor recovery alone, but also applies to cognitive impairments such as visuospatial neglect and aphasia [28]. This latter finding suggests that the current recommendations for designing trials are probably generalizable to other modalities affected after stroke.

The following points should be taken into account when interpreting the results. First, generalizability of the current results may be limited because the estimates were derived from a single study of a relatively small population of patients with first-ever ischemic hemispheric stroke. Despite this small population, the heterogeneity between patients in the current study is representative for other stroke populations [6, 12, 32]. Second, we only included two important factors for designing an RCT in the present study. Other important factors that should be taken into account are, for example: the biological rationale behind the research protocol including selection of research interventions and dose of therapy, and selecting the appropriate outcome measures [33, 34]. Third, the prognostic stratification that was used in the original RCT was based on the first assessment at approximately 1 week after stroke. We used the SDs determined for different time intervals as a representation for the SDs at 26-week follow-up. We did not, however, account for possible changes in prognosis for upper limb capacity over time (i.e., return of VFE). As there were a number of patients in the group without VFE at intake who showed more recovery than expected based on their prognosis early after stroke, the SDs for the first few time intervals in the group of patients without VFE may not be completely representative for the SD at 26-week follow-up. Fourth, the estimated sample sizes in the first few time intervals with the ARAT were very small (Tables 2 and 3). The estimated sample sizes were even smaller in solely the group of patients without VFE. These results point out the considerable impact of prognostic stratification and time between stroke onset and randomization in RCTs. However, we do not recommend that researchers design RCTs with these very small numbers of participants as they will be prone to error. Fifth, for modeling purposes we assumed a normally distributed outcome and performed a standard *t* test sample size calculation, commonly used in stroke trials. If the distribution of the outcome was not normally distributed, a rank-based test may have been more appropriate. Sixth, a sample size estimation based on a standard *t* test may overestimate the sample size compared to a stratified version of the test or a regression model. Higher power may be attained when stratification is included in the analysis stage.

## Conclusion

The timing of the moment of randomization post stroke, and stratification based on the prognostic determinant VFE, are fundamental for designing upper limb trials early post stroke. To increase the chance of finding differential intervention effects, future RCTs should randomize patients at fixed moments after stroke and stratify patients according to their potential neurobiological recovery.

## Abbreviations

ARAT: Action Research Arm Test
EMG-NMS: EMG-triggered Neuro-muscular Stimulation
FMA-UE: Fugl-Meyer Assessment Upper Extremity
MCID: Minimal Clinically Important Difference
mCIMT: Modified Constraint-induced Movement Therapy
NIHSS: National Institutes of Health Stroke Scale
RCT: Randomized controlled trial
VFE: Voluntary Finger Extension
